# Cerebral infarct induced by severe leptospirosis-a case report and literature review

**DOI:** 10.1186/s12883-022-03021-5

**Published:** 2022-12-29

**Authors:** Zhongli Zhu, Jian Feng, Yong Dong, Bin Jiang, Xiong Wang, Fuxiang Li

**Affiliations:** 1 Department of critical care medicine, The General Hospital of Western Theater Command, 270 Tianhui Road, Chengdu, Sichuan 610083 China; 2 Department of critical care medicine, Dazhu County People’s Hospital, 99 Qingnian Road, Sichuan Dazhou, 635199 China; 3 Department of cardiovascular medicine, The General Hospital of Western Theater Command, 270 Tianhui Road, Chengdu, Sichuan 610083 China

**Keywords:** Case report, Leptospirosis, Cerebral infarct, Next-generation sequencing (NGS), Microscopic agglutination test (MAT)

## Abstract

**Background:**

Although most leptospirosis is mild, the severe form can cause multiple complications, with a fatality rate of over 50% even with ICU support. The clinical manifestations of leptospirosis vary depending on organs and tissues involved. Both cerebral artery and coronary artery can be damaged by leptospirosis. Although cerebral arteritis induced by leptospirosis has been reported, cerebral infarction caused by leptospirosis is rarely reported.

**Case presentation:**

We report the case of a 79-year-old man admitted to intensive care unit (ICU) because of 3 days duration of fever, bloody sputum and dyspnea. Five days before he was admitted to hospital, he had harvested rice in flooded fields. After admission, leptospira interrogans DNA sequence was identified in bronchoalveolar lavage fluid (BALF) by next-generation sequencing (NGS). Microscopic agglutination test (MAT) showed the serum antibody of Mini serovars was 1,600 and Hebdomadis serovars was 800. On the eighth day of admission, the patient noted left hemiplegia. Cranial CT scan revealed low-density shadow in the right basal ganglia, so cerebral infarction was diagnosed. The patient’s condition rapidly deteriorated and he died on the eleventh day of admission despite penicillin treatment, invasive mechanical ventilation and continuous renal replacement support.

**Conclusion:**

Neurologic leptospirosis manifested as cerebral occlusion, although rare, might be deadly and should not be ignored.

**Supplementary Information:**

The online version contains supplementary material available at 10.1186/s12883-022-03021-5.

## Background

Leptospirosis, also called Weil’s disease, is a zoonotic infectious disease caused by pathogenic strains of the bacterium Leptospira. Although it has a worldwide distribution, it is especially prevalent in east/south-east Asia and South America [1]. Although most leptospirosis is mild, severe types of the disease can cause acute respiratory distress syndrome (ARDS), acute hepatic failure, acute kidney injury (AKI), and multiple organ dysfunction syndrome (MODS) [2]. The case-fatality rate of severe leptospirosis can rise to 52% even with ICU support [3]. It is estimated that annually there are 1.03 million cases and 58,900 deaths attributed to leptospirosis worldwide, with resource-poor countries having the highest morbidity and mortality, particularly in regions where the burden of leptospirosis has been underappreciated [4].

The clinical manifestations of leptospirosis are complicated because of the difference of impaired organs or tissues. Generally, the clinical signs and symptoms of leptospirosis include chills, high fever, weakness, headache, myalgia (especially gastrocnemius tenderness), superficial lymphadenectasis, and conjunctival congestion [5]. Leptospirosis has a biphasic course of illness. The first phase corresponds to the multiplication and spread of the organism throughout the body, and the second phase is characterized by the development of circulating antibodies and the detection of leptospires in the urine. In the second phase, the clinical signs of leptospirosis are more organ specific, which include hemoptysis/blood sputum, dyspnea, oliguria, jaundice, and uveitis [6]. The disease can be categorized into anicteric and icteric forms, and the icteric form is the more severe form, which accounts for 5-10% of patients [7]. Neuroleptospirosis, which is often manifested by aseptic meningitis, intracerebral bleed, spinal extradural hematoma, myelopathy, myeloradiculopathy, Guillain-Barré syndrome, neuropathies (mononeuritis, facial palsy), and myositis, is usually unexplored and overlooked [8–10]; but to our knowledge, cerebral infarction because of leptospirosis is rarely reported [11]. In this report, we are able to identify an uncommon case of cerebral infarction caused by leptospirosis.

## Case presentation

**Fig. 1:**
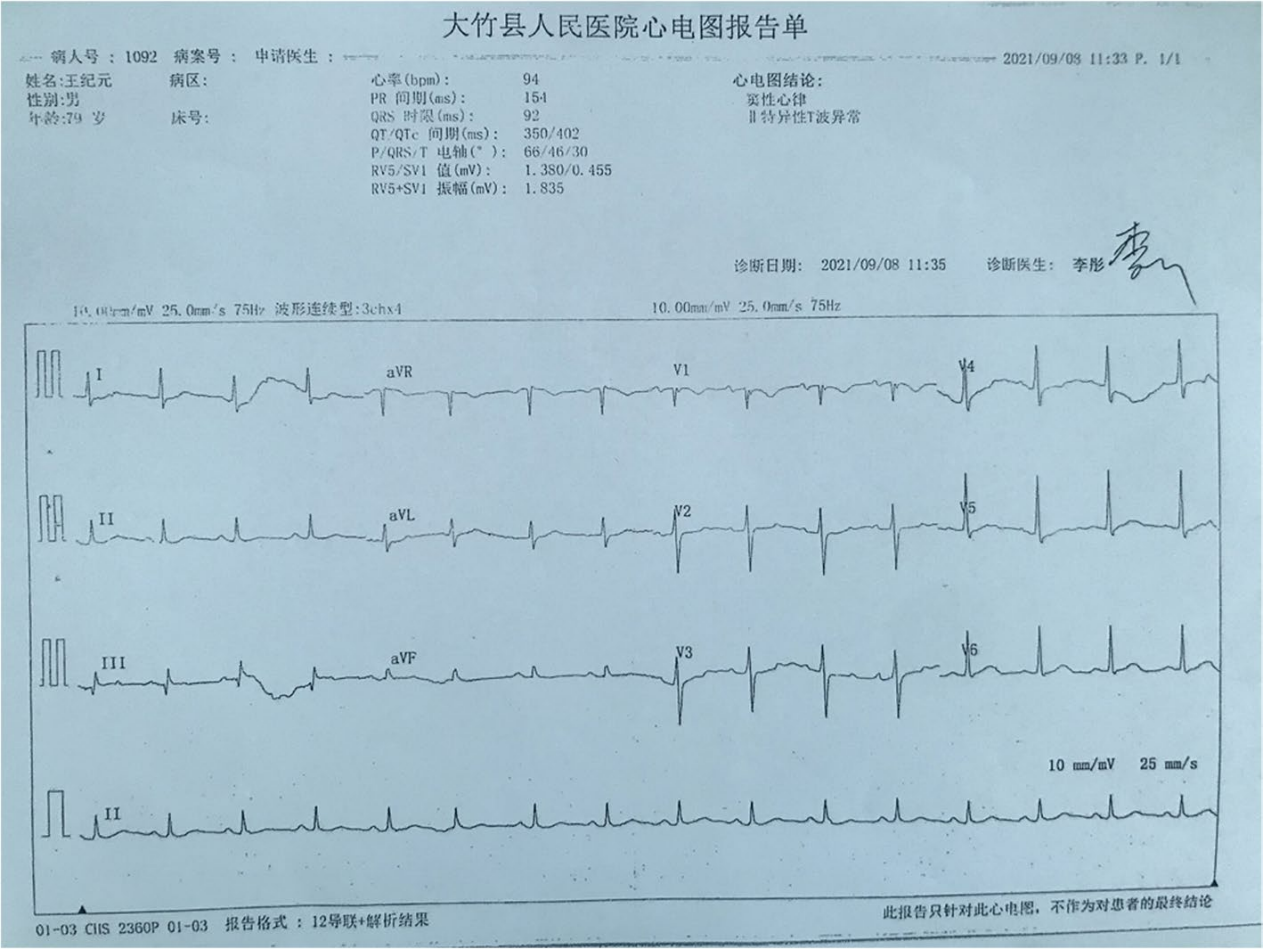
On September 8, 2021, electrocardiogram showed: (1) Sinus rhythm; (2) T wave anomaly

**Fig. 2:**
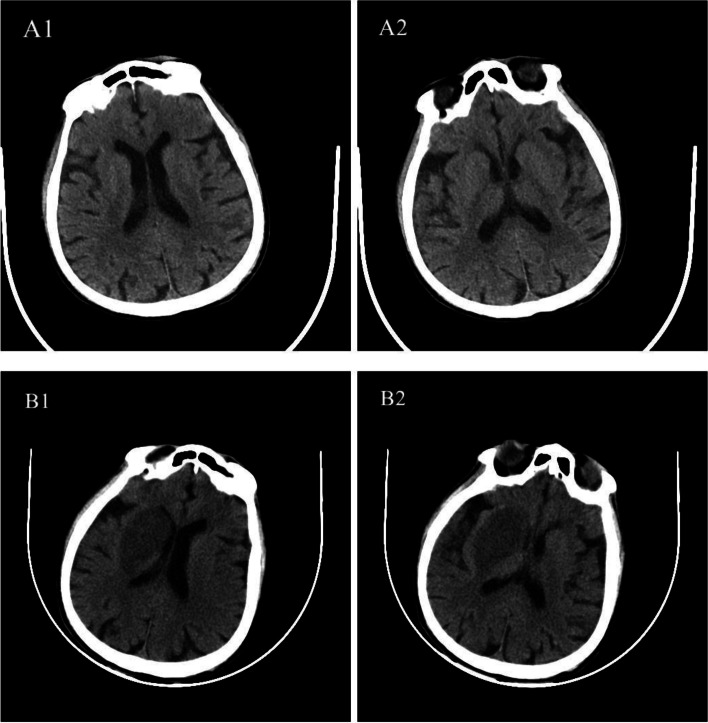
Series head CT scan. A1-A2 scan obtained on illness days 8 showed a normal head CT. B1-B2 scan obtained on illness days 11 showed a low-density shadow in the right basal ganglia

On September 8, 2021, a 79-year-old man was admitted to the respiratory medicine department with a complaint of 3 days duration of fever, chills, bloody sputum and dyspnea. He was a rural farmer and there was a flooding event because of a heavy rain in his hometown 7 days before the onset. 2 days after the heavy rain, the patient harvested the rice in the paddy field. The patient had been well until 3 days before admission, when a moderate fever, chills and a cough productive of bloody sputum developed. The patient received antibiotic treatment of levofloxacin in a simple clinic in his hometown two days after the onset, but got no improvement in fever. Moreover, the symptom of dyspnea was getting worse. After the first treatment at the local clinic, the patient was hospitalized in the respiratory department of our hospital. The patient reported no history of headache or myalgia and denied having hypertension, diabetes, arrhythmia, coronary heart disease and tumor, which is consistent with blood pressure, laboratory findings, electrocardiogram (ECG, Fig. 1), cardiac ultrasound (Supplementary Fig. 1), abdominal ultrasound (Supplementary Fig. 2), thorax-CT (Supplementary Fig. 3A1-A3) and head CT (Fig. 2A1-A2) during hospitalization.

After 1 day of hospitalization in the respiratory department, the patient was referred to the intensive care unit (ICU) because of severe community acquired pneumonia with respiratory failure. On arrival at the ICU, the patient was very sick and drowsy. The patient presented tachycardia 135 beats per minute, body temperature 36℃, shortness of breath 35 times per minute, blood pressure 82/47mmHg, and oxygen saturation 75% while breathing ambient air. Jaundice was observed. Moist rales could be heard in both lungs. The extremities were cold, and the mottling score was 3. The superficial lymph nodes were not palpable. There was no gastrocnemius tenderness. The remainder of the examination was normal. The arterial blood gas analysis showed the following values: potential of hydrogen (PH) 7.44, partial pressure of carbon dioxide (PCO_2_) 24.6 mmHg, partial pressure of oxygen (PO_2_) 37 mmHg(fraction of inspiration O2 45%), actual bicarbonate radical (HCO_3_) -16.7 mmol/L, base excess (BE) -6 mmol/L, lactic acid (Lac) 3.8 mmol/L. The initial laboratory test revealed leukocytosis with white blood cell (WBC) 19.98 × 10^9^/L, neutrophils accounting for 89.5%, thrombocytopenia with 72 × 10^9^ platelets/L, hypersensitive C-reactive protein (hs-CRP): 199.66 mg/L, procalcitonin (PCT): 99.572 ng/ml, serum creatinine(Scr): 150.00 umol/L, total bilirubin (TBI): 63.60 umol/L, direct bilirubin (DBI): 43.50 umol/L, and Alanine aminotransferase (ALT) and glutamic oxalacetic transaminase (AST) at 30.20 IU/L and 76.80 IU/L, respectively. An emergency chest high resolution computed tomography (HRCT) scan was performed, and the results revealed both lungs had patchy ground glass opacity (GGO), a finding suggestive of pneumonia (Fig. 1A1-A3).

Accordingly, the primary diagnosis was made as follows: (1) severe community acquired pneumonia, (2) septic shock, (3) acute respiratory distress syndrome, and 4)multiple organ dysfunction syndrome. The patient received treatment with tracheal intubation, invasive mechanical ventilation and fluid resuscitation(sodium lactate ringer’s injection, 30ml / kg for 2 hours [12]). Intravenous antibiotics (levofloxacin, 500 mg, q.d.; Imipenem/Cilastatin Sodium, 1,000 mg, t.i.d.) were administered empirically. The fever gradually eased and the oxygen saturation improved. The blood and sputum cultures for bacteria and fungi were sterile. On September 13, a head CT scan was obtained which appeared normal (Fig. 2A1-A2) and the second chest CT scan showed most of the pulmonary ground glass opacity absorbed (Supplementary Fig. 3B1-B3). The bedside bronchoscopy showed a little bloody mucous sputum in the bronchi. According to the patient’s clinical manifestation and laboratory test results, we highly suspected he must be infected by some unknown pathogenic microorganism. On the 5th day after admission, we employed next-generation sequencing technique to detect etiology in the bronchoalveolar lavage fluid. Leptospira interrogans deoxyribonucleic acid (DNA) sequences were identified by next-generation sequencing analysis two days later. Then, we sent his blood to local disease control and prevention center to verify the next-generation sequencing results and the results of serum antibody titre tested by microscopic agglutination test were as follows: Leptospirosis interrogans serogroup Mini serovar for 1,600 and Leptospirosis interrogans serogroup Hebdomadis serovar for 800. We found the patient presented with left upper and lower limb mobility disorder on September 15, and muscle strength was grade 0. A second head CT scan (Fig. 2B1-B2) was performed on September 16th, and the results showed cerebral atrophy and a low-density shadow in the right basal ganglia. Compared with the first head CT scan on September 13th (Fig. 2A1-A2), the low-density shadow in the right basal ganglia was a new lesion. Therefore, the final diagnosis was as follows: (1) leptospirosis, (2) septic shock, (3) acute respiratory distress syndrome, (4) multiple organ dysfunction syndrome, and (5) Cerebral infarct. The patient was treated with intravenous penicillin 0.4 mU, q8h on September 16th. 7 h after the first dosage of penicillin, the patient’s condition deteriorated, with an abrupt fever of 40℃ and oxygen saturation dropped to 82%. The patient was considered to have developed Herxheimer reaction and was immediately given sedatives and intravenous hydrocortisone. Thereafter, the patient experienced a period of decreasing body temperature and increasing oxygen saturation. Nevertheless, the patient’s condition deteriorated further, requiring continuous pumping of norepinephrine to maintain blood pressure. Two days later, the patient died due to sepsis-induced acute circulation failure.

## Discussion and conclusions

The first human leptospirosis case in China was reported in 1937 and leptospirosis has caused several epidemics in China in the last century [13]. So far, more than 2.4 million cases and over 20,000 deaths due to leptospirosis have been reported in China [13]. The incidence of leptospirosis has dramatically decreased in recent years, probably due to the lower leptospire-carrying rate in pigs [14].

Previously reported in the literature, the neurological leptospirosis often presents with meningitis and clouded sensorium in the early phase and typical neurological manifestations that include headache, vomiting, and meningeal irritation in the late phase [15]. Other neurological manifestations include myeloradiculopathy, myelopathy, Guillain-Barré syndrome like presentation, meningoencephalitis, intracerebral bleed, cerebellar dysfunction, iridocyclitis and tremor/rigidity [16]. In 1975, Chinese researchers from Wuhan Medical College first reported that cerebral occlusion was due to cerebral arteritis in a leptospirosis endemic area in China where the patients had a history of contact with pigs or contaminated water [16]. They conducted three autopsy cases and found the pathological changes were basically the same inflammatory cerebral arteritis and there were leptospiral bacteria on the cerebral artery wall in one case [16]. Another four cases of leptospirosis post mortem study showed cerebral arteritis involving predominantly large arteries at the base of the brain, especially the middle and anterior cerebral arteries [17]. Furthermore, it seems leptospirosis can damage not only the cerebral artery, but also the coronary artery [18, 19]. A review of cerebral infarction caused by intracranial infection suggested that a minority of leptospirosis patients may develop intracranial arterial disease [20]. In these cases, cerebral panarteritis involves the main trunks of larger arteries at the base of the brain, and watershed and anterior circulation infarctions may occur [21]. But as far as we know, cerebral infarction caused by leptospirosis is rarely reported. This is a rare leptospirosis case report, which was complicated with cerebral infarction during the early stage of the disease. Based on previous studies and head CT scan results, we speculate that the cerebral infarction of our patient was likely caused by intracranial artery disease induced by leptospirosis. Therefore, we emphasize that the possibility of cerebral infarction caused by leptospirosis should be considered in the treatment of leptospirosis.

Diagnosis of leptospirosis depends on simple diagnostic tests, which are often ignored because of a low index of clinical suspicion. Uncommon manifestation of leptospirosis, especially when presented as an acute neurological manifestation, often poses a diagnostic dilemma leading to delayed treatment and the potential for adverse clinical outcomes [22, 23]. We made the same mistake until next-generation sequencing was employed which detected the unknown pathogenic microorganism in the lung. After that, the sequences of Leptospira interrogans were identified in the bronchoalveolar lavage fluid. next-generation sequencing has the same principle of gene amplification as PCR. PCR, which has been suggested to be an early diagnostic method for leptospirosis in several literatures, can only detect known sequences [15, 24, 25]. However, next-generation sequencing is a hypothesis-free approach that does not require prior knowledge of sequence information and has the ability to sequence millions of fragments simultaneously per run. A previous case report of neuroleptospirosis suggested that next-generation sequencing coupled with a rapid bioinformatics pipeline provided a clinically actionable diagnosis of a specific infectious disease from an uncommon pathogen [26]. Hence, next-generation sequencing is increasingly used in clinics for determining unknown etiologic infections in China. Beyond that, microscopic agglutination test is the reference standard test for serological diagnosis of leptospires and has high sensitivity and specificity [27]. According to the criteria made by the “Leptospirosis Reference Epidemiology Group” (LERG), a confirmed case of leptospirosis should have an acute MAT titre of ≥ 1 in 400 or a four-fold rise of titre between acute and convalescent samples. A probable case is defined when the MAT titre is ≥ 1 in 100 in a non-endemic area [28]. This case showed the antibody titre detected by the microscopic agglutination test method on the 10th day of admission as follows: Leptospirosis interrogans serogroup Mini serovar for 1,600 and Leptospirosis interrogans serogroup Hebdomadis serovar for 800. Although there was no comparison between Mini serovar and Hebdomadis serovar, the titre of antibody was so high that we could draw the conclusion of leptospirosis. Notably, the serum contained a high level antibody of two types of leptospira serovar, both of which belong to the Leptospira interrogans serogroup. We speculated that the patient may in fact be simultaneously infected by two types of bacterium Leptospira since the patient lived in an area where it is endemic and both serovars are common pathogens [29]. Therefore, the possibility that two types of bacterium leptospira had the same cross antigen could not be excluded [25]. Consequently, for severe patients with atypical symptoms but suspected infectious disease, we recommend that eligible hospitals should perform next-generation sequencing testing. Then, according to the results of next-generation sequencing, the examination and treatment should be further improved. Once leptospirosis is diagnosed, mild cases may not require antibiotics. However, appropriate therapy with intravenous penicillin or ceftriaxone should be initiated empirically for severe disease. The role of steroids in the treatment of leptospirosis phase is controversial [30, 31]. Based on our clinical experience, we believe that steroid hormones should be considered in the management of critically ill patients. Furthermore, since the prognosis of neuroleptospirosis is largely unknown, we recommend routine treatment of cerebral infarction on the basis of systemic treatment of leptospirosis.

In conclusion, leptospirosis is a global zoonosis caused by pathogenic strains of the bacterium Leptospira. It is often presented with atypical clinical manifestations, which makes diagnosis challenging. Clinicians practising in tropical or endemic regions should consider leptospirosis infection in patients presenting with acute fever of unknown cause. Neurologic leptospirosis manifested as cerebral occlusion, although rare, might be deadly and should not be ignored.

## Supplementary information

**Additional file 1: Supplementary Fig 1. **Cardiac ultrasound on September 10, 2021 showed: 1. Right heart enlargement; 2. Severe mitral regurgitation and mild pulmonary hypertension; 3. Left ventricular systolic function is acceptable.

**Additional file 2: Supplementary Fig 2. **Abdominal ultrasound on September 10, 2021 showed: there were no abnormalities in the liver, gallbladder, pancreas, spleen and kidney

**Additional file 3: Supplementary Fig 3. **Series chest high resolution CT scans during his stay in the hospital. A1-A3 scan obtained on illness days 3 showed bilateral patchy ground-glass opacities in both lungs. B1-B3 scan obtained on illness days 8 showed most of the ground glass opacity absorbed. C1-C3 scanobtained on illness days 11 showed apparent infiltration in both lungs, predominantly in the lower parts of lungs.

## Data Availability

The datasets used and/or analyzed during the current study are available from the corresponding author on reasonable request.

## Abbreviations

ICU: intensive care unit
BALF: broncho alveolar lavage fluid
DNA: deoxyribonucleic acid
NGS: next-generation sequencing
CT: computed tomography
HRCT: high resolution CT
MAT: microscopic agglutination test
ARDS: acute respiratory distress syndrome
AKI: acute kidney injury
MODS: multi-organ dysfunction
GGO: ground glass opacities
PCT: procalcitonin
CRP: C-reactive protein
Scr: serum creatinine
TBI: total bilirubin
DBI: direct bilirubin
