# Supplementary figures and images for: Cerebral infarct induced by severe leptospirosis-a case report and literature review
Source: BMC Neurol. 2022 Dec 29;22:506. doi: 10.1186/s12883-022-03021-5 (PMC9798630; doi:10.1186/s12883-022-03021-5)

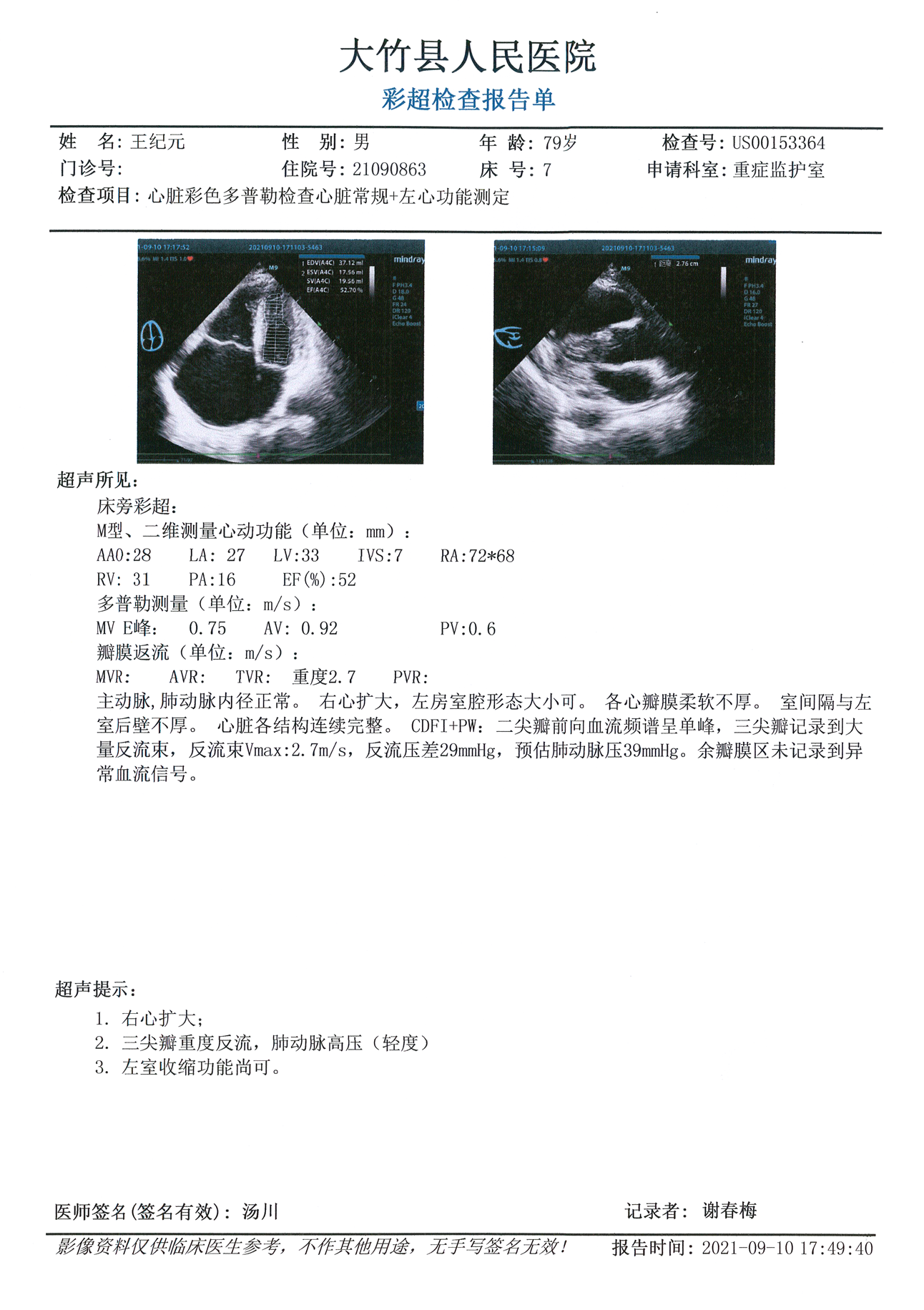

Supplement: Supplementary file 1 — Additional file 1: Supplementary Fig 1. Cardiac ultrasound on September 10, 2021 showed: 1. Right heart enlargement; 2. Severe mitral regurgitation and mild pulmonary hypertension; 3. Left ventricular systolic function is acceptable. [file 12883_2022_3021_MOESM1_ESM.tif]

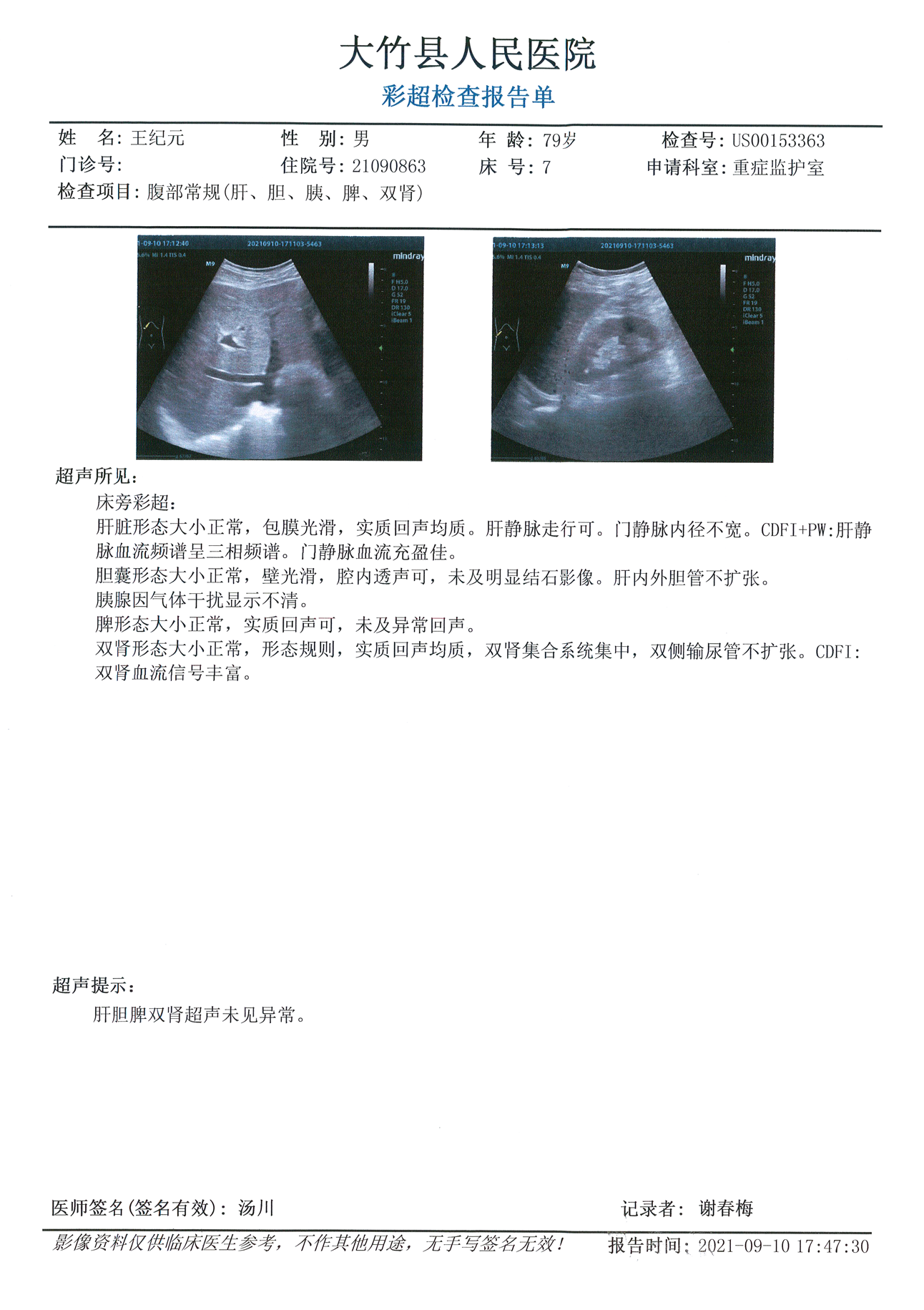

Supplement: Supplementary file 2 — Additional file 2: Supplementary Fig 2. Abdominal ultrasound on September 10, 2021 showed: there were no abnormalities in the liver, gallbladder, pancreas, spleen and kidney [file 12883_2022_3021_MOESM2_ESM.tif]

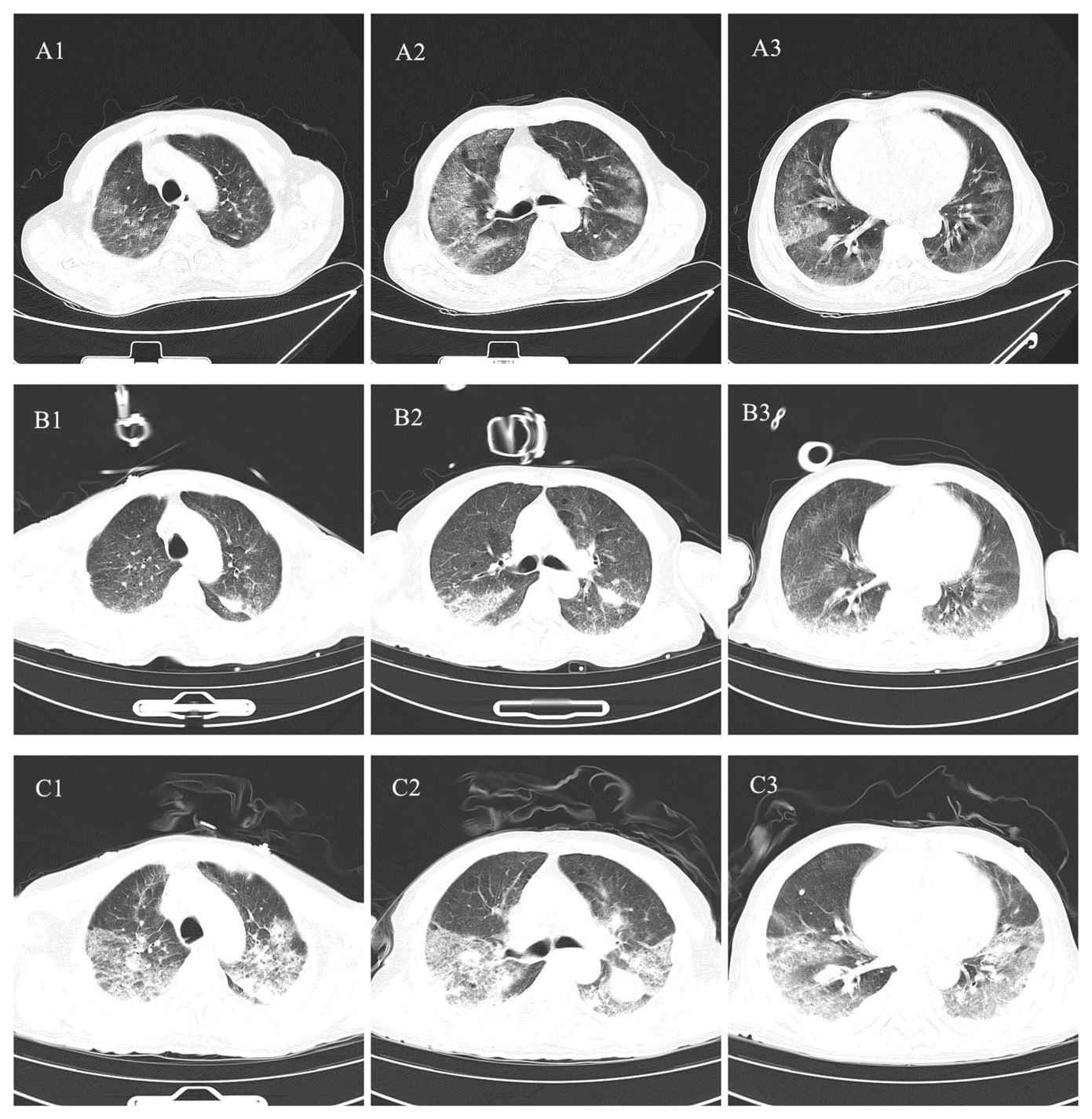

Supplement: Supplementary file 3 — Additional file 3: Supplementary Fig 3. Series chest high resolution CT scans during his stay in the hospital. A1-A3 scan obtained on illness days 3 showed bilateral patchy ground-glass opacities in both lungs. B1-B3 scan obtained on illness days 8 showed most of the ground glass opacity absorbed. C1-C3 scanobtained on illness days 11 showed apparent infiltration in both lungs, predominantly in the lower parts of lungs. [file 12883_2022_3021_MOESM3_ESM.tif]
